# Supplementary material for: Inverse altitude effect disputes the theoretical foundation of stable isotope paleoaltimetry
Source: Nat Commun. 2022 Jul 28;13:4371. doi: 10.1038/s41467-022-32172-9 (PMC9334263; doi:10.1038/s41467-022-32172-9)
Supplement: Supplementary file 1 — Supplementary Information [file 41467_2022_32172_MOESM1_ESM.pdf]

Supplementary Information for

# Inverse altitude effect disputes the theoretical foundation of stable isotope paleoaltimetry

Zhaowei Jing[1,2,3], Wusheng Yu[1,*], Stephen Lewis[4], Lonnie G. Thompson[5], Jie Xu[1,6], Jingyi Zhang[1,6], Baiqing Xu[1], Guangjian Wu[1], Yaoming Ma[1], Yong Wang[1,6], Rong Guo[1,6]

[1]State Key Laboratory of Tibetan Plateau Earth System, Resources and Environment (TPESRE), Institute of Tibetan Plateau Research, Chinese Academy of Sciences, Beijing 100101, China.

[2]Deep-Sea Multidisciplinary Research Center, Pilot National Laboratory of Marine Science and Technology (Qingdao), 266237 Qingdao, China.

[3]Frontiers Science Center for Deep Ocean Multi-spheres and Earth System, Key Laboratory of Physical Oceanography, Ocean University of China, 266100 Qingdao, China.

[4]Catchment to Reef Research Group, Centre for Tropical Water and Aquatic Ecosystem Research, James Cook University, Townsville, QLD 4811, Australia.

[5]Byrd Polar and Climate Research Center, The Ohio State University, Columbus, OH 43210, USA

[6]University of Chinese Academy of Sciences, Beijing, China.

*Corresponding author. Email: yuws@itpcas.ac.cn

**This PDF file includes:**

Supplementary Figs. 1 to 13

Supplementary Tables 1 to 3

Supplementary References

**Supplementary Figs.**

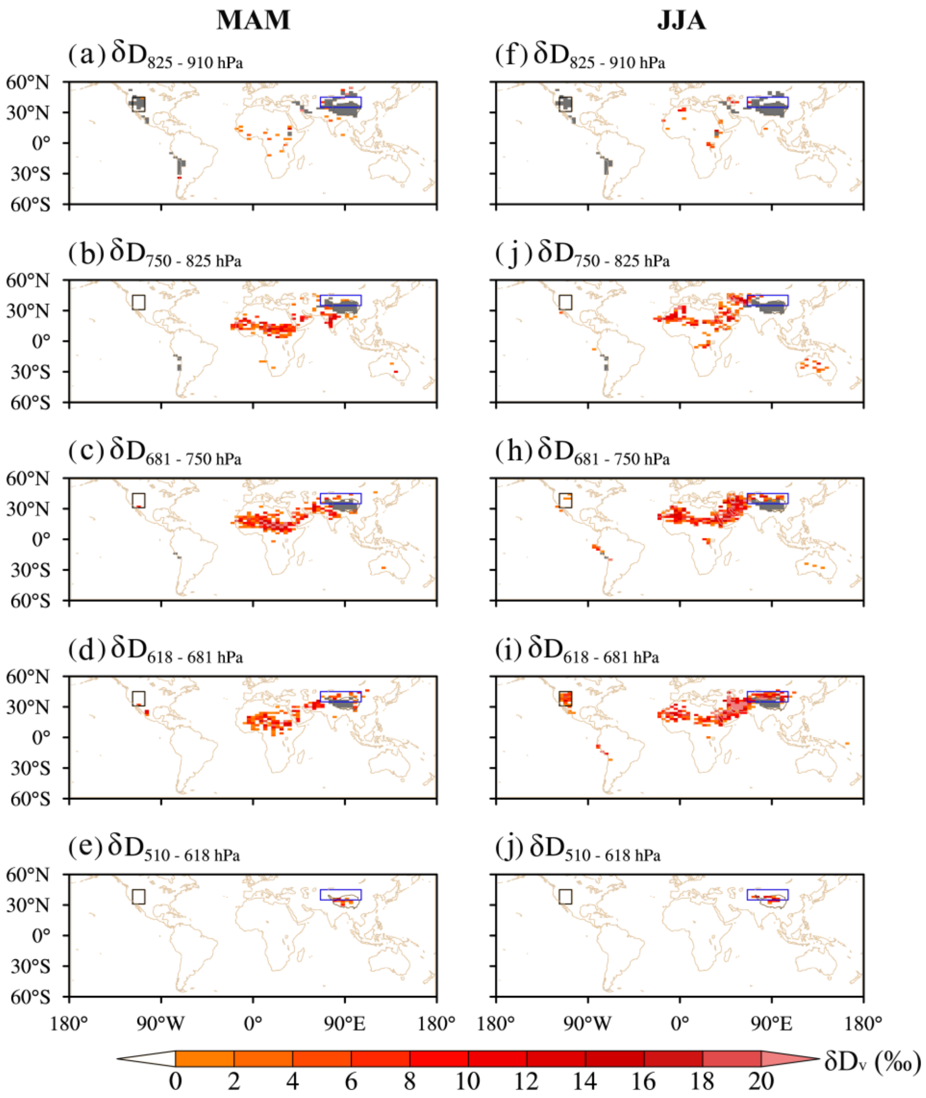


**Supplementary Fig. 1. Seasonal spatial patterns of the IAE at different atmospheric levels at the lower and mid-latitudes. a-e** spring (March-April-May: MAM). **f-j** summer (June-July-August: JJA). The black boxes show the WUSA region (31° N – 45° N, 118° W – 106° W) while the blue boxes indicate the NTP region (35° N – 45° N, 66° E – 106° E). The IAE is very weak across the globe under the 825 hPa level. Hence, in this study we mainly discuss the IAE above the 825 hPa level. Gray shading indicates that no valid $\delta D_v$ data are available for the specific grid. IAE, inverse altitude effect. WUSA, western United States of America. NTP, northern Tibetan Plateau. This map was generated with The NCAR Command Language (Version 6.6.2) [Software]. (2019). Boulder, Colorado: UCAR/NCAR/CISL/TDD. http://dx.doi.org/10.5065/D6WD3XH5.

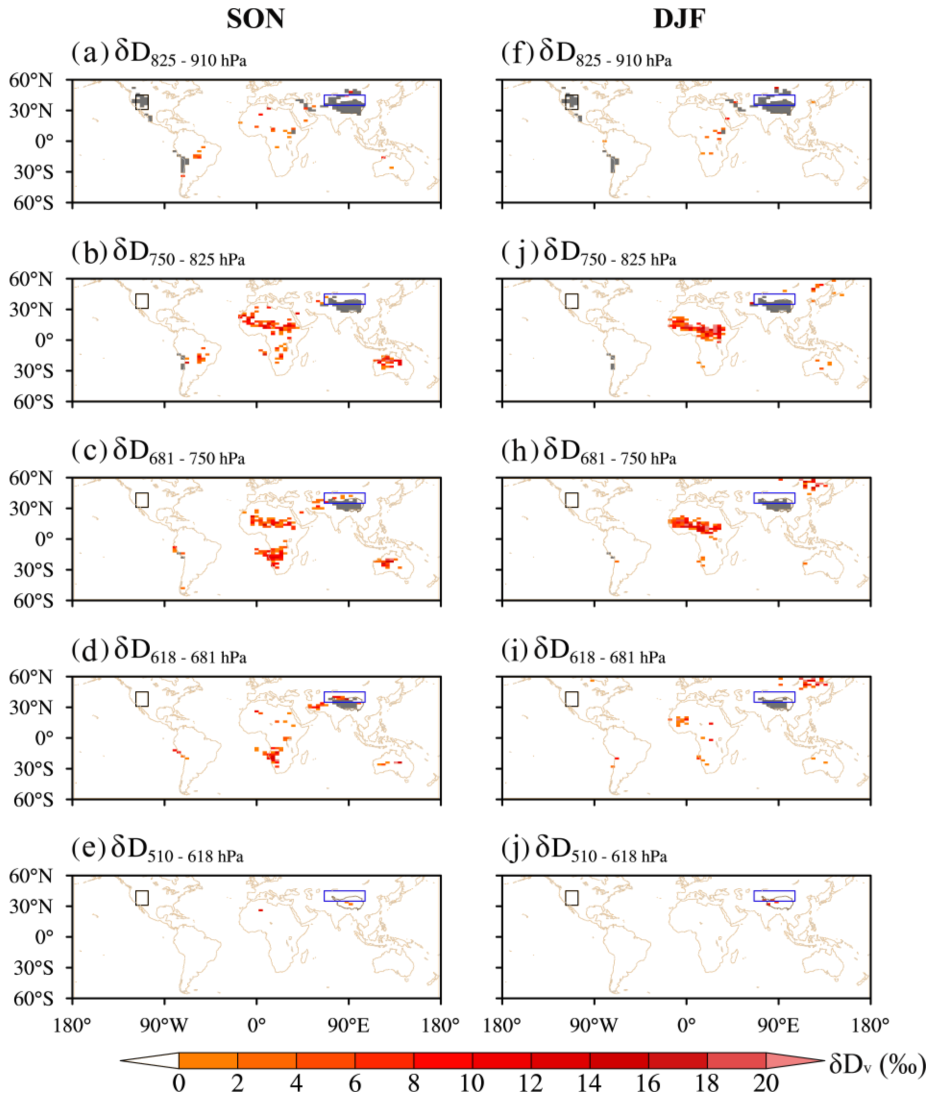


**Supplementary Fig. 2. Seasonal spatial patterns of the IAE at different atmospheric levels at the lower and mid-latitudes. a-e** autumn (September-October-November: SON). **f-j** winter (December-January-February: DJF). The black boxes show the WUSA region (31° N – 45° N, 118° W – 106° W) while the blue boxes indicate the NTP region (35° N – 45° N, 66° E – 106° E). The IAE is very weak under the 825 hPa level across the globe. Hence, in this study we mainly discuss the IAE above the 825 hPa level. Gray shading indicates that no valid $\delta D_v$ data are available for the specific grid. IAE, inverse altitude effect. WUSA, western United States of America. NTP, northern Tibetan Plateau. This map was generated with The NCAR Command Language (Version 6.6.2) [Software]. (2019). Boulder, Colorado: UCAR/NCAR/CISL/TDD. http://dx.doi.org/10.5065/D6WD3XH5.

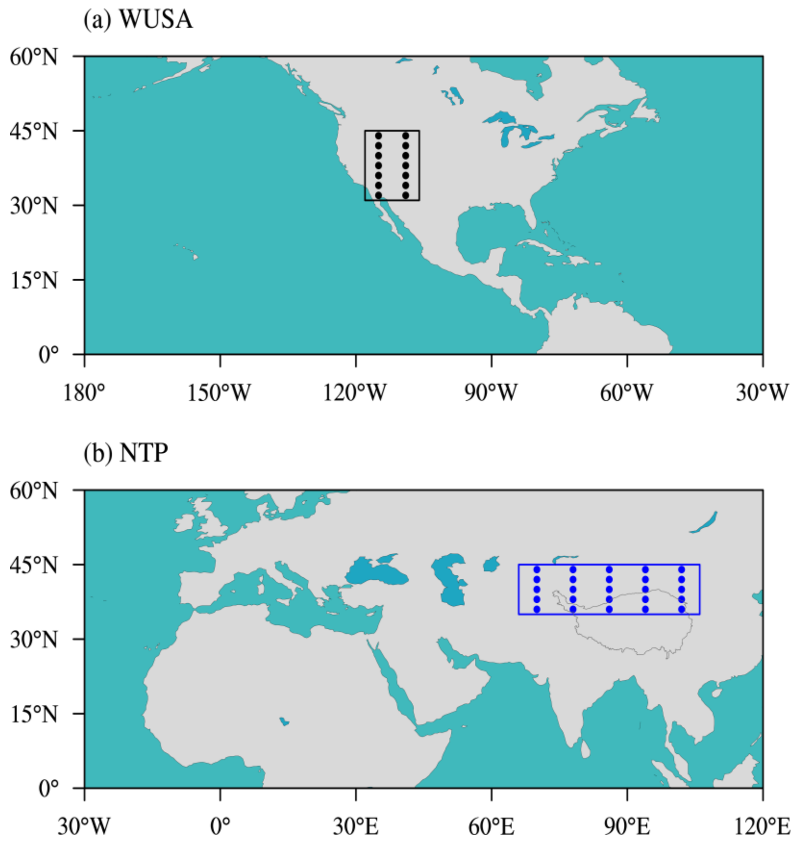


**Supplementary Fig. 3. Trajectory starting positions. a** Geographical distribution of trajectory starting positions (black dots) for the WUSA. **b** Geographical distribution of trajectory starting positions (blue dots) for the NTP. Gray line in panel **b** indicates the boundary of the Tibetan Plateau. IAE, inverse altitude effect. WUSA, western United States of America. NTP, northern Tibetan Plateau. This map was generated with The NCAR Command Language (Version 6.6.2) [Software]. (2019). Boulder, Colorado: UCAR/NCAR/CISL/TDD. http://dx.doi.org/10.5065/D6WD3XH5.

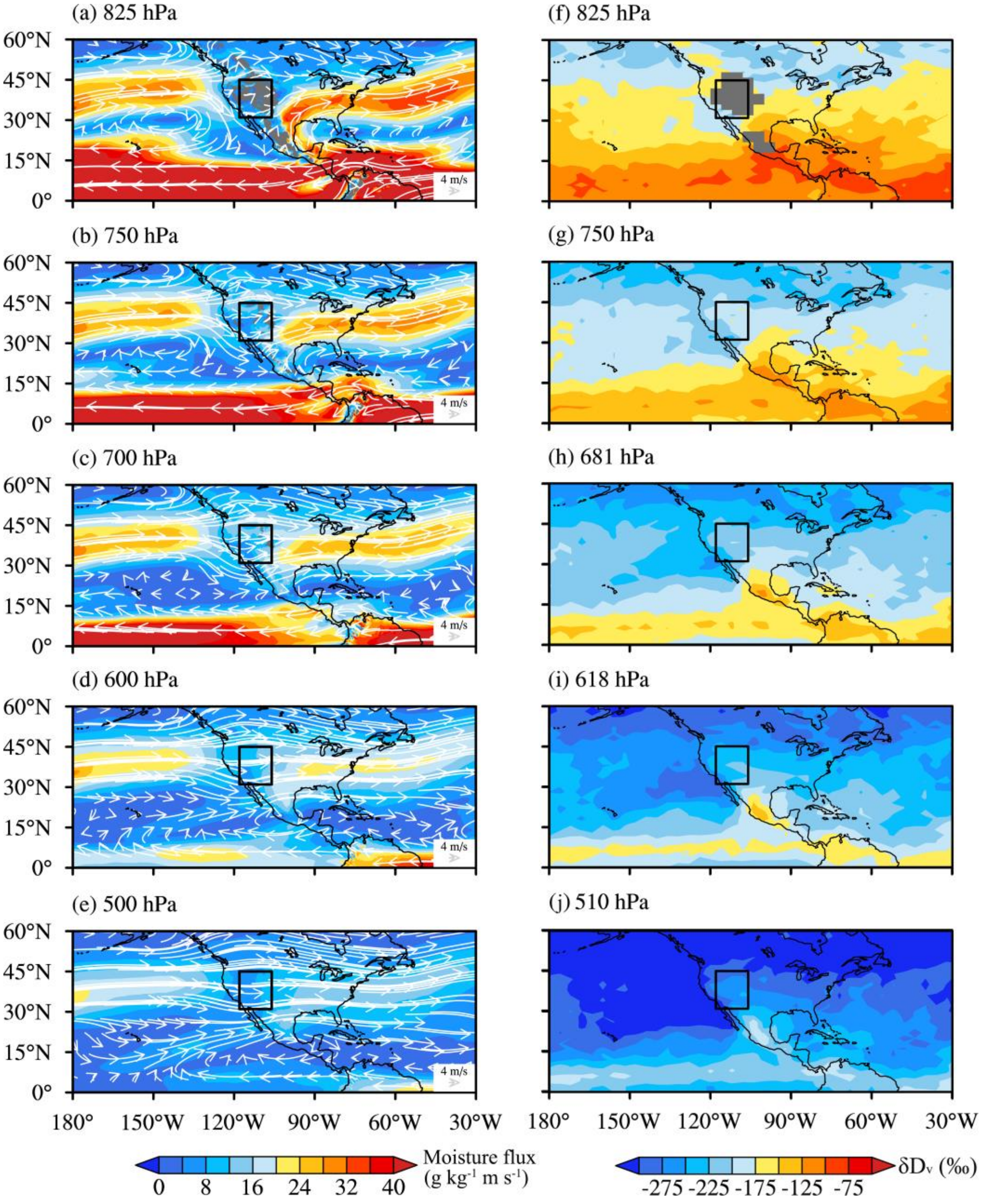


**Supplementary Fig. 4. Atmospheric circulation patterns and δD$_v$ during spring (March-April-May: MAM) over North America and surroundings during 2006-2009. a-e** Moisture fluxes (shading) and wind fields (vector) at different atmospheric levels based on ERA5 reanalysis (left panels). **f-j** δD$_v$ at the different atmospheric levels based on TES retrievals (right panels). Gray shading in panels **a**, **b**, **c**, and **f** indicates that no valid data are available for the specific grid. This map was generated with The NCAR Command Language (Version 6.6.2) [Software]. (2019). Boulder, Colorado: UCAR/NCAR/CISL/TDD. http://dx.doi.org/10.5065/D6WD3XH5.

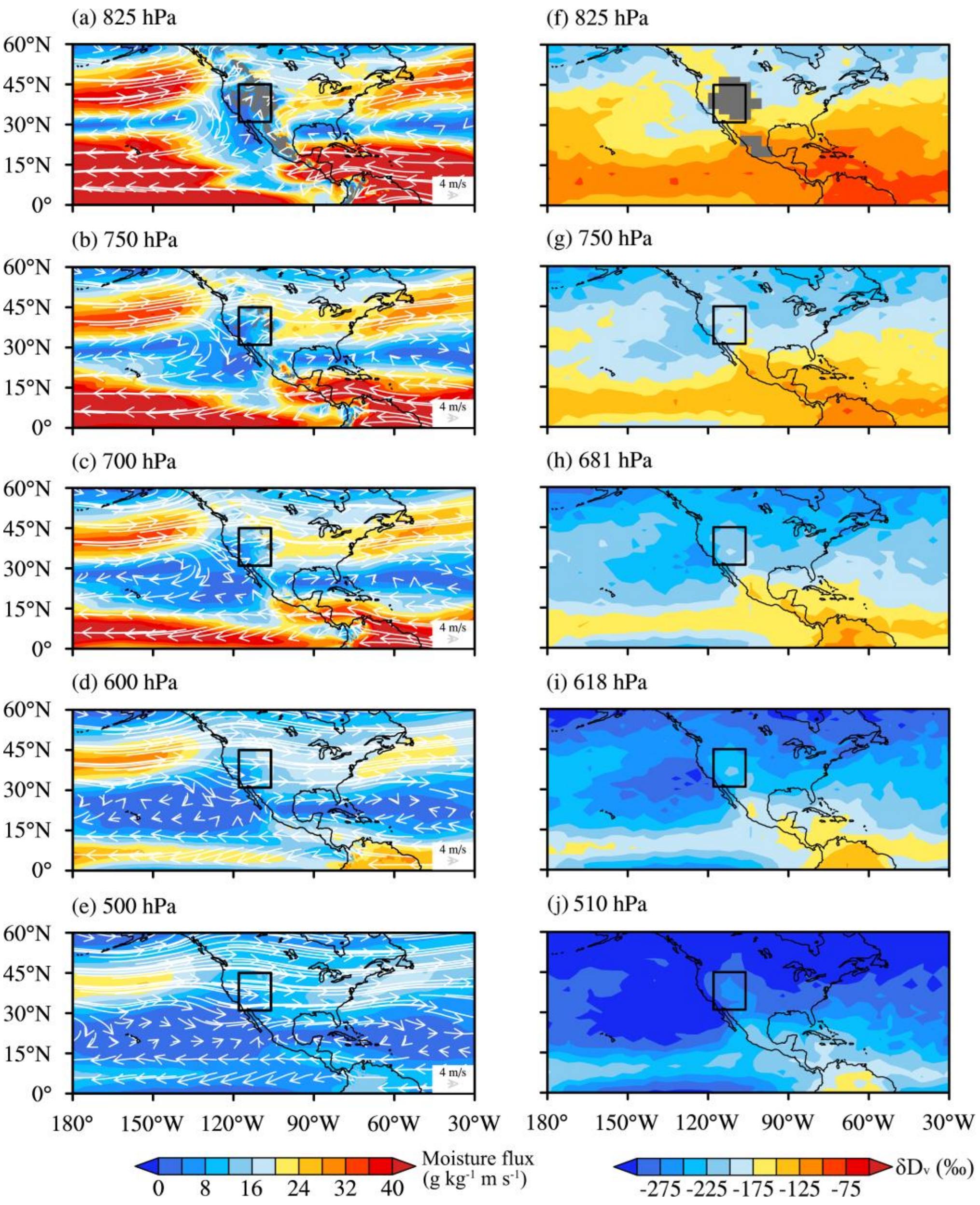


**Supplementary Fig. 5. Atmospheric circulation patterns and $\delta D_v$ during autumn (September-October-November: SON) over North America and surroundings during 2006-2009. a-e** Moisture fluxes (shading) and wind fields (vector) at different atmospheric levels based on ERA5 reanalysis (left panels). **f-j** $\delta D_v$ at the different atmospheric levels based on TES retrievals (right panels). Gray shading in panels **a**, **b**, **c**, and **f** indicates that no valid data are available for the specific grid. This map was generated with The NCAR Command Language (Version 6.6.2) [Software]. (2019). Boulder, Colorado: UCAR/NCAR/CISL/TDD. http://dx.doi.org/10.5065/D6WD3XH5.

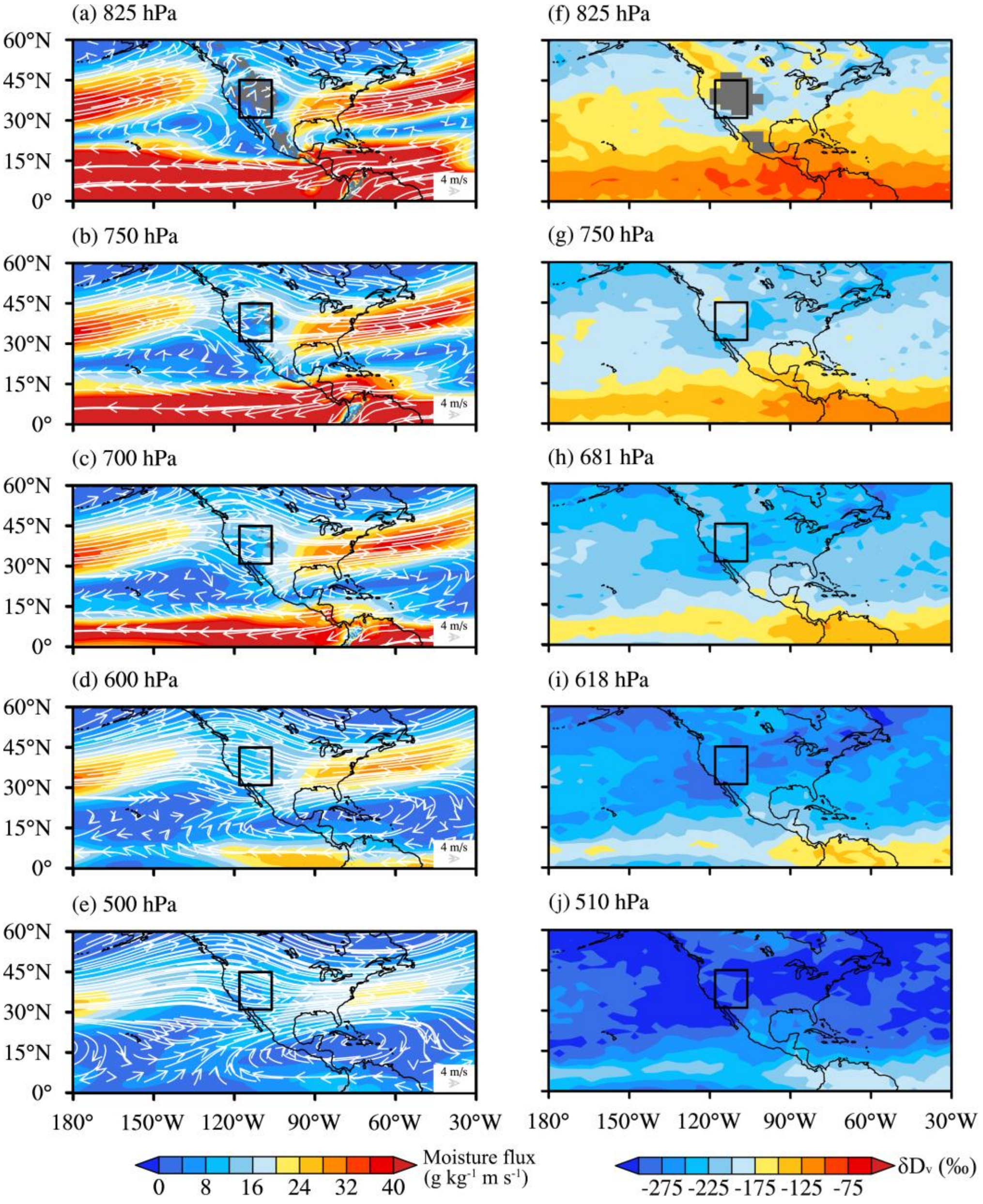


**Supplementary Fig. 6. Atmospheric circulation patterns and $\delta D_v$ during winter (December-January-February: DJF) over North America and surroundings during 2006-2009. a-e** Moisture fluxes (shading) and wind fields (vector) at different atmospheric levels based on ERA5 reanalysis (left panels). **f-j** $\delta D_v$ at the different atmospheric levels based on TES retrievals (right panels). Gray shading in panels **a**, **b**, **c**, and **f** indicates that no valid data are available for the specific grid. This map was generated with The NCAR Command Language (Version 6.6.2) [Software]. (2019). Boulder, Colorado: UCAR/NCAR/CISL/TDD. http://dx.doi.org/10.5065/D6WD3XH5.

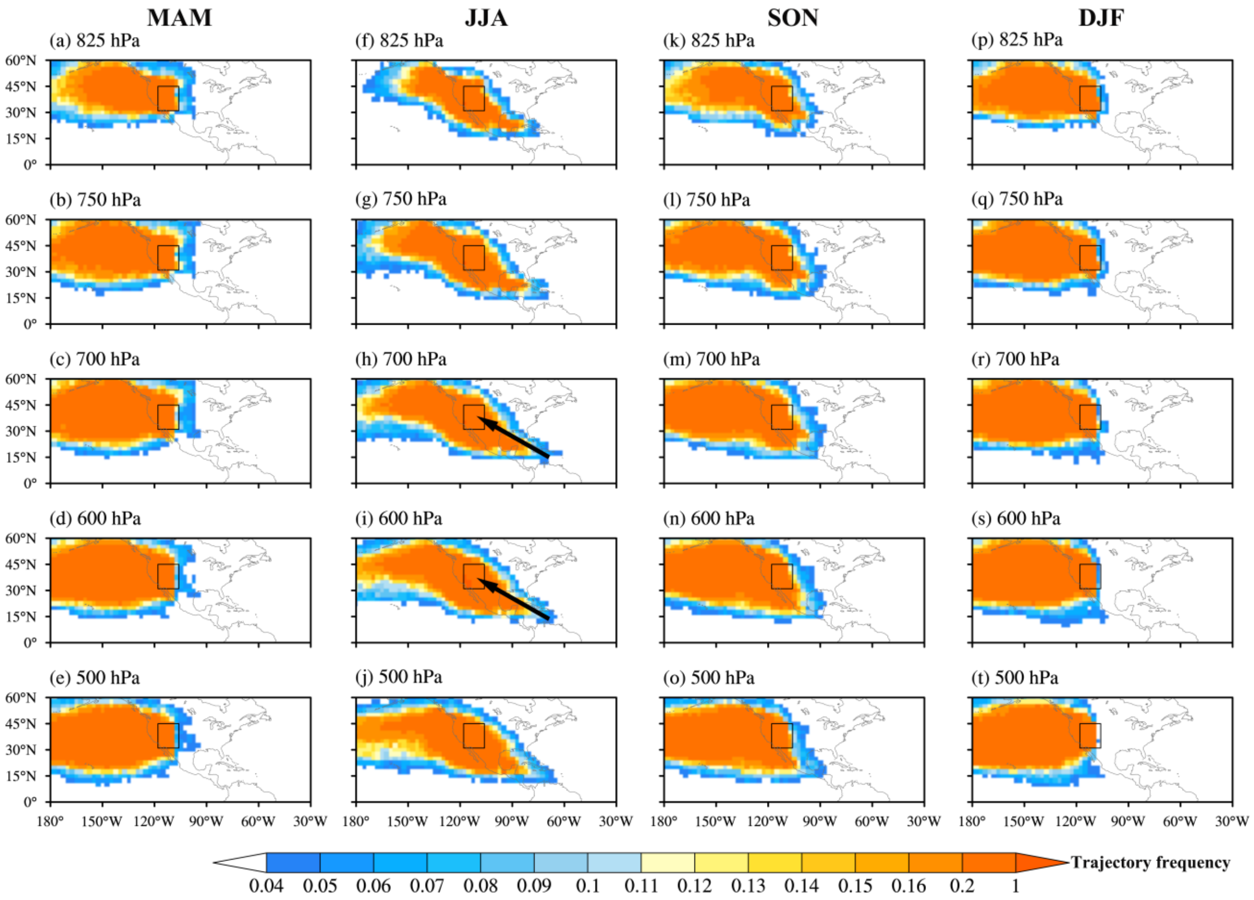


**Supplementary Fig. 7. Seasonal trajectory frequencies for different atmospheric levels in the WUSA during 2006-2009. a-e** spring (March-April-May: MAM). **f-j** summer (June-July-August: JJA). **k-o** autumn (September-October-November: SON). **p-t** winter (December-January-February: DJF). WUSA, western United States of America. This map was generated with The NCAR Command Language (Version 6.6.2) [Software]. (2019). Boulder, Colorado: UCAR/NCAR/CISL/TDD. http://dx.doi.org/10.5065/D6WD3XH5.

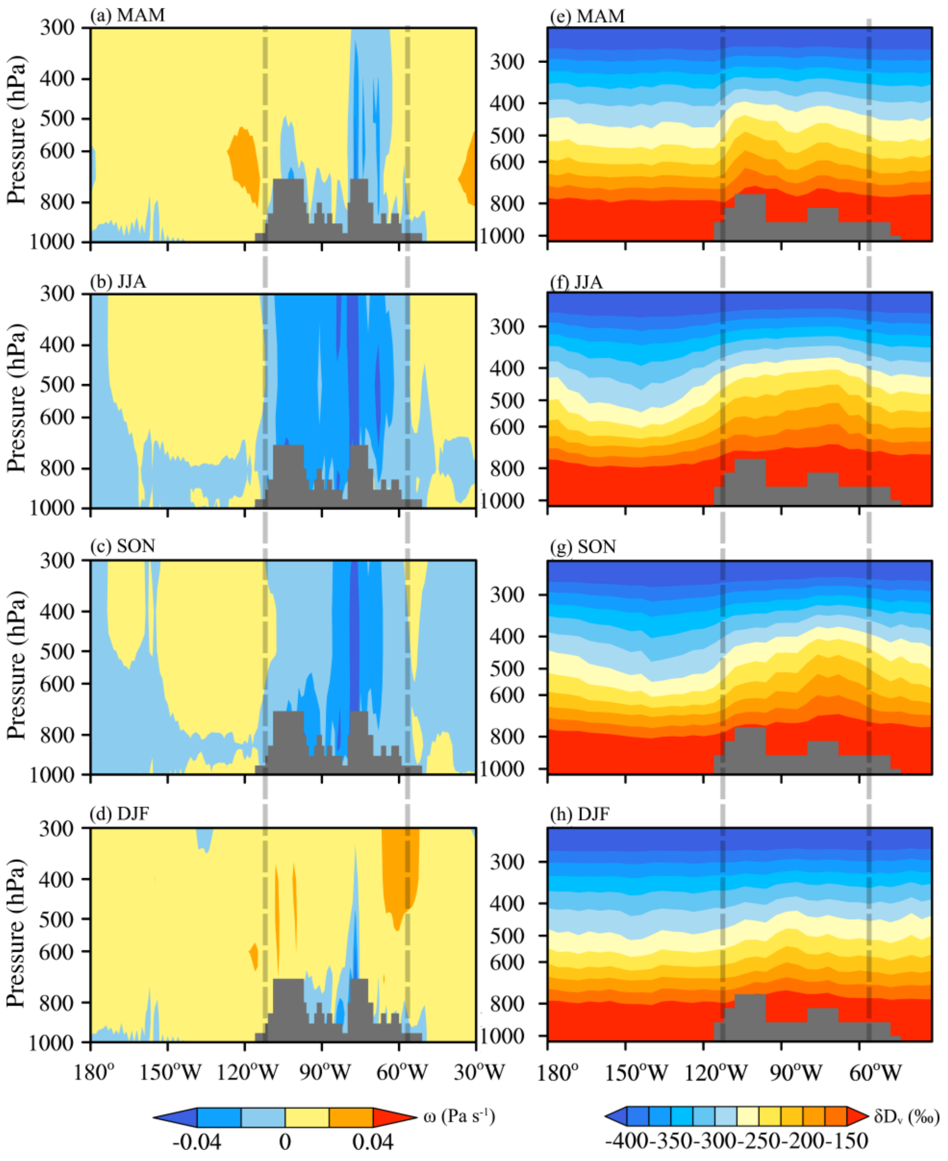


**Supplementary Fig. 8. Seasonal vertical profiles of meridional average (0°N – 30°N) vertical velocities and $\delta D_v$ over the tropical Atlantic and surroundings during 2006-2009. a-d** Vertical velocities based on ERA5 reanalysis (left panels). **e-h** $\delta D_v$ based on TES retrievals (right panels). The dashed lines represent the longitudinal range of the area where the vertical upward movement is strongest in summer (June-July-August: JJA). The gray shading in each panel represents the highest elevation of the surface within a range of longitude, reflecting the complexity of the terrain in each area.

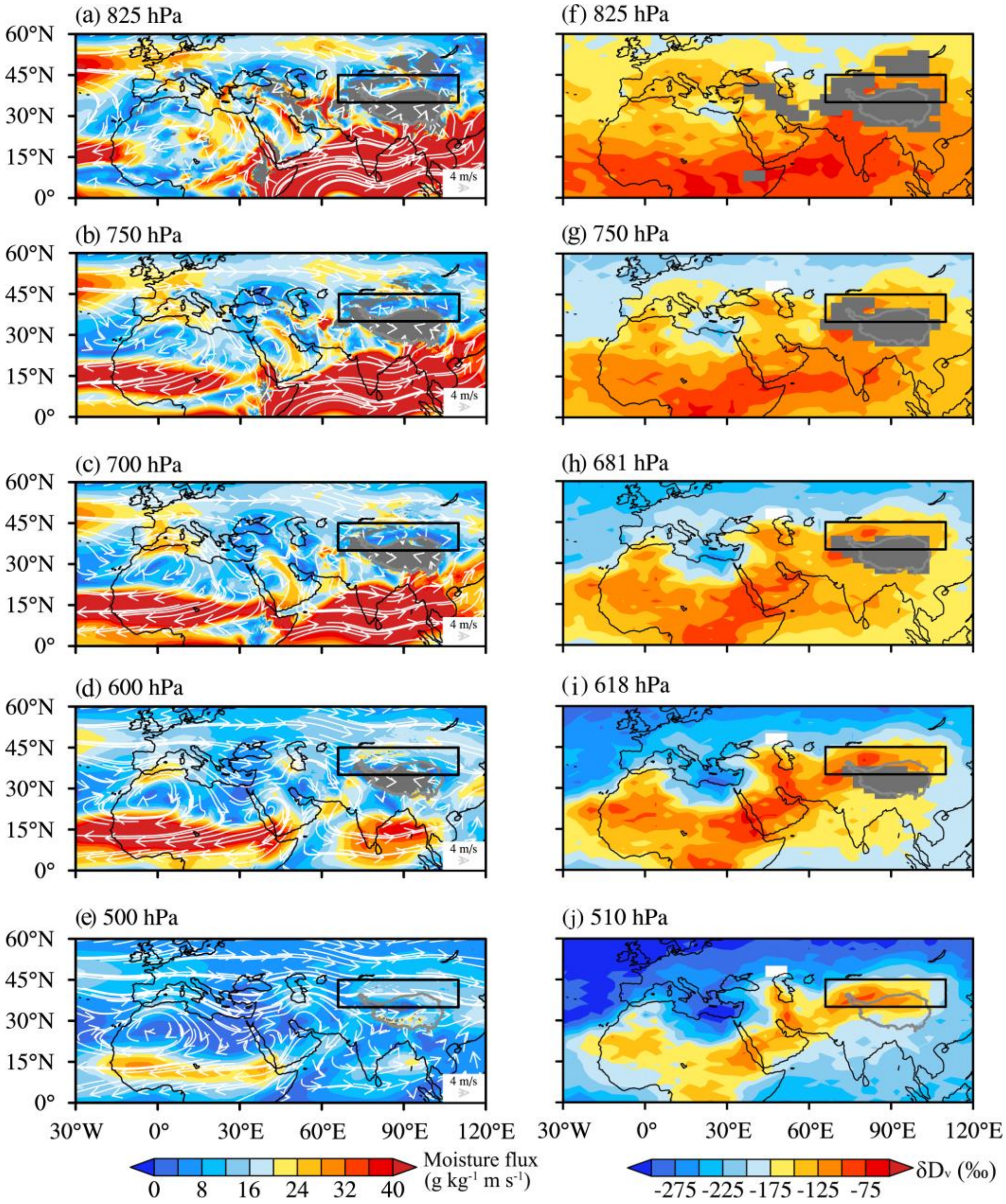


**Supplementary Fig. 9. Atmospheric circulation patterns and $\delta D_v$ during summer (June-July-August: JJA) over the Asian drylands and surroundings during 2006-2009. a-e** Moisture fluxes (shading) and wind fields (vector) at different atmospheric levels based on ERA5 reanalysis (left panels). **f-j** $\delta D_v$ at the different atmospheric levels based on TES retrievals (right panels). Gray shading in each panel indicates that no valid data are available for the specific grid. This map was generated with The NCAR Command Language (Version 6.6.2) [Software]. (2019). Boulder, Colorado: UCAR/NCAR/CISL/TDD. http://dx.doi.org/10.5065/D6WD3XH5.

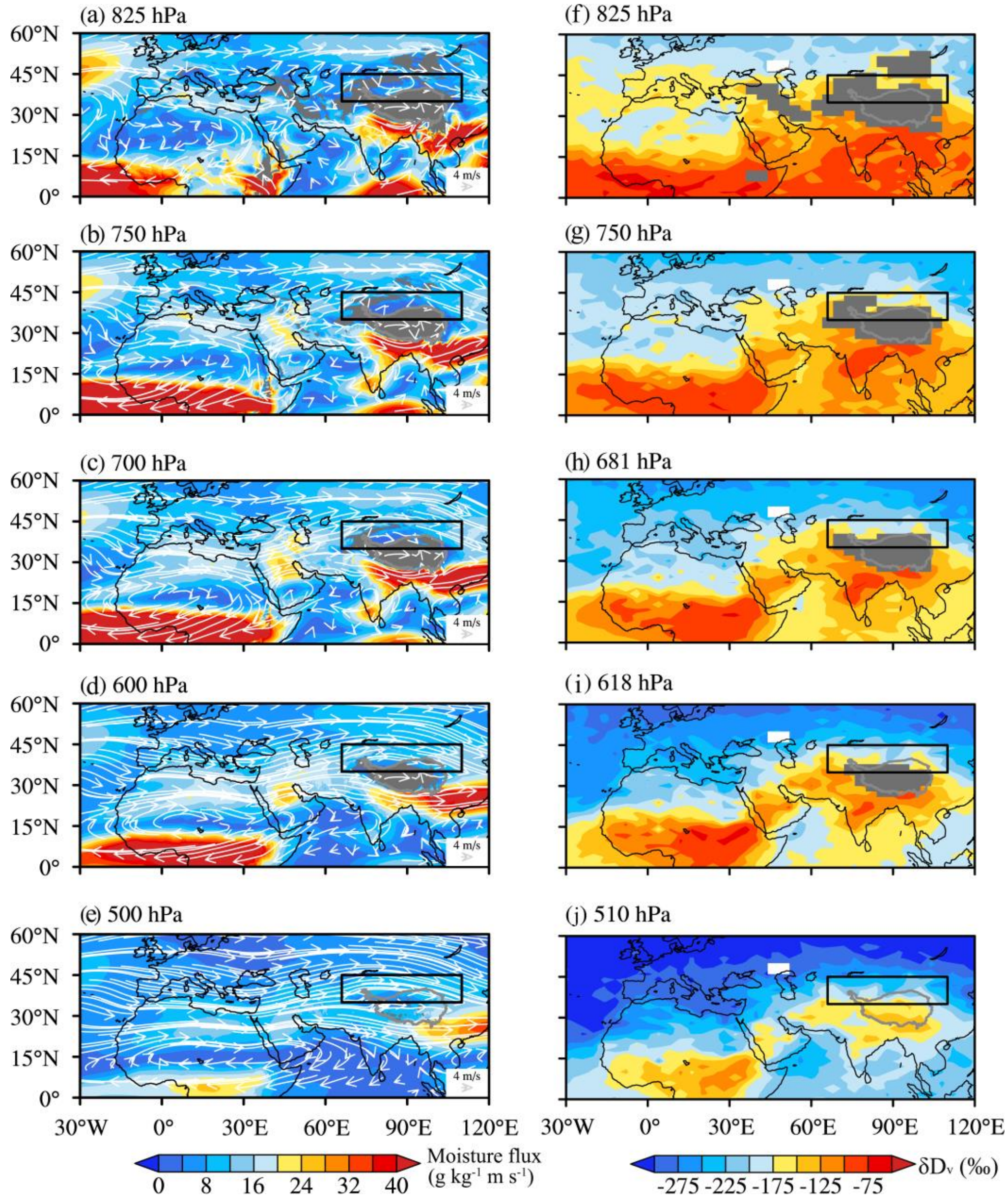


**Supplementary Fig. 10. Atmospheric circulation patterns and $\delta D_v$ during spring (March-April-May: MAM) over the Asian drylands and surroundings during 2006-2009. a-e** Moisture fluxes (shading) and wind fields (vector) at different atmospheric levels based on ERA5 reanalysis (left panels). **f-j** $\delta D_v$ at the different atmospheric levels based on TES retrievals (right panels). Gray shading in each panel indicates that no valid data are available for the specific grid. This map was generated with The NCAR Command Language (Version 6.6.2) [Software]. (2019). Boulder, Colorado: UCAR/NCAR/CISL/TDD. http://dx.doi.org/10.5065/D6WD3XH5.

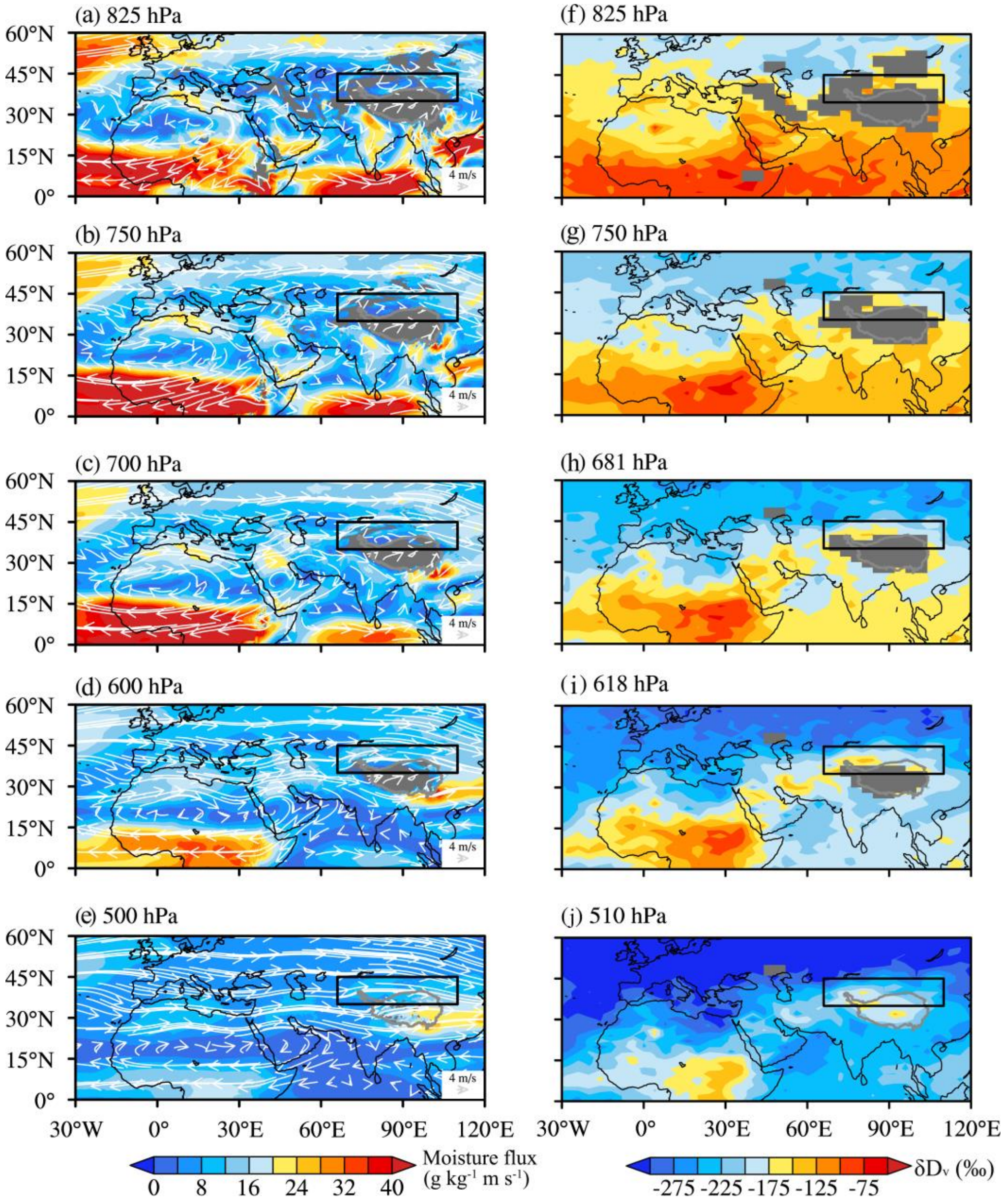


**Supplementary Fig. 11. Atmospheric circulation patterns and δD$_v$ during autumn (September-October-November: SON) over the Asian drylands and surroundings during 2006-2009. a-e** Moisture fluxes (shading) and wind fields (vector) at different atmospheric levels based on ERA5 reanalysis (left panels). **f-j** δD$_v$ at the different atmospheric levels based on TES retrievals (right panels). Gray shading in each panel indicates that no valid data are available for the specific grid. This map was generated with The NCAR Command Language (Version 6.6.2) [Software]. (2019). Boulder, Colorado: UCAR/NCAR/CISL/TDD. http://dx.doi.org/10.5065/D6WD3XH5.

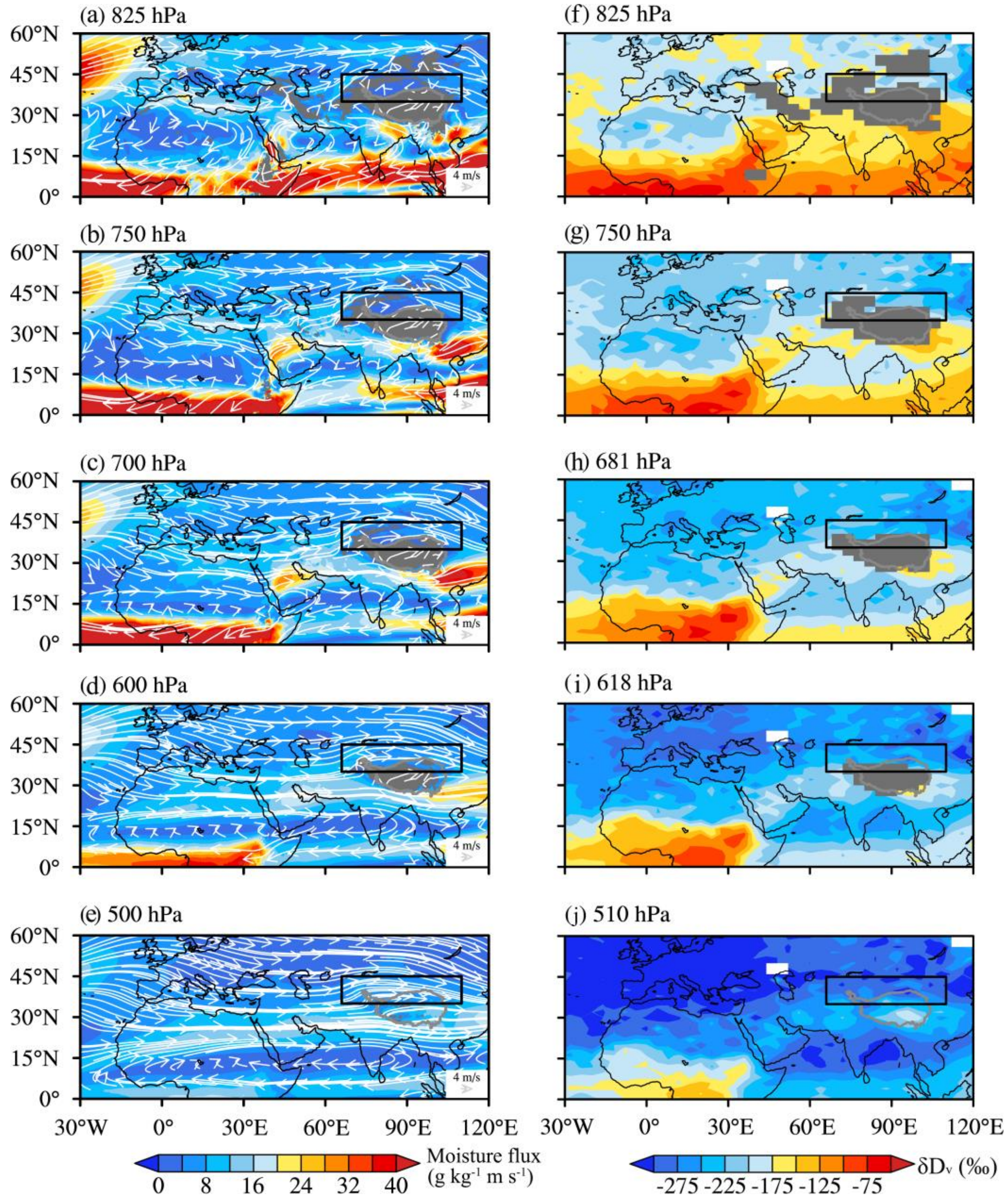


**Supplementary Fig. 12. Atmospheric circulation patterns and δDv during winter (December-January-February: DJF) over the Asian drylands and surroundings during 2006-2009. a-e** Moisture fluxes (shading) and wind fields (vector) at different atmospheric levels based on ERA5 reanalysis (left panels). **f-j** $\delta D_v$ at the different atmospheric levels based on TES retrievals (right panels). Gray shading in each panel indicates that no valid data are available for the specific grid. This map was generated with The NCAR Command Language (Version 6.6.2) [Software]. (2019). Boulder, Colorado: UCAR/NCAR/CISL/TDD. http://dx.doi.org/10.5065/D6WD3XH5.

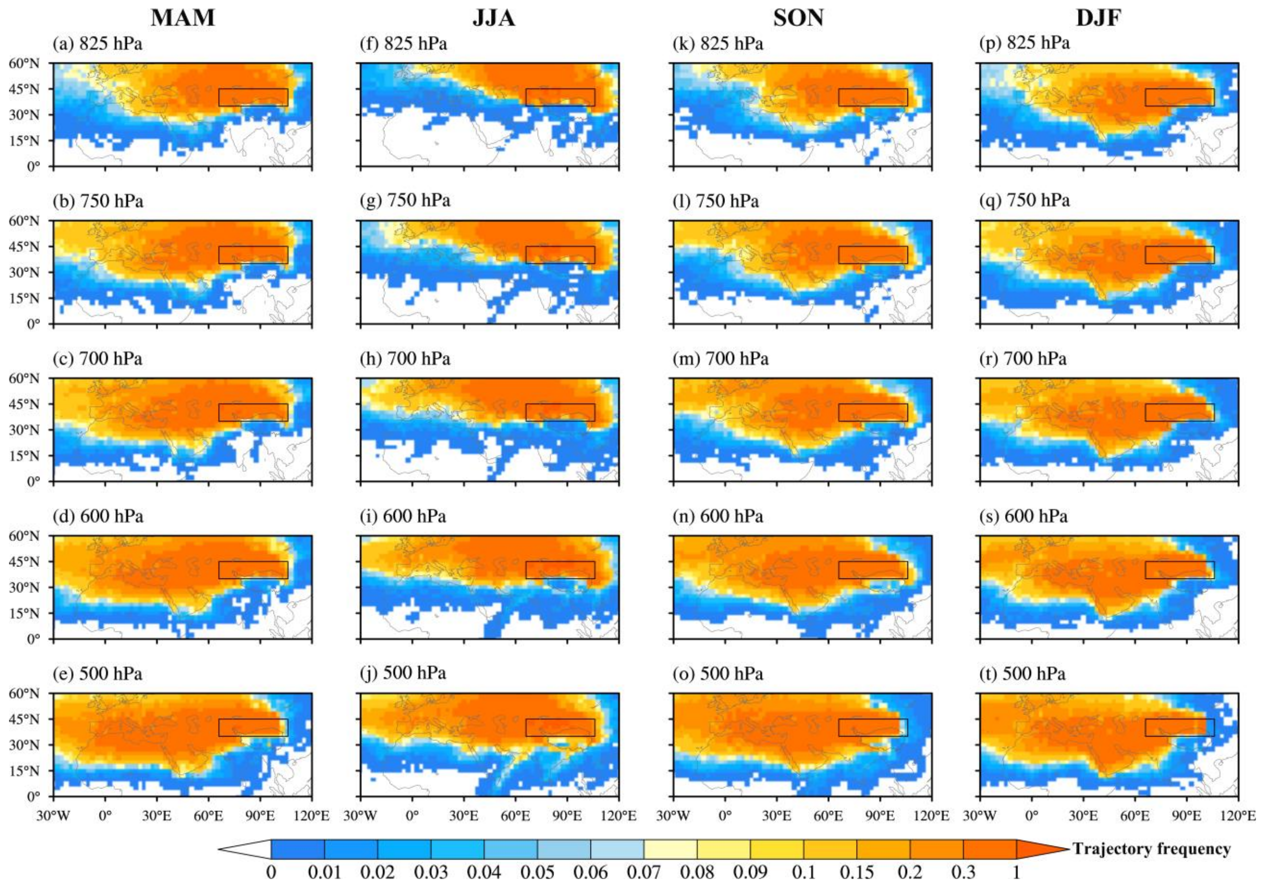


**Supplementary Fig. 13. Seasonal trajectory frequency for different atmospheric levels in the NTP during 2006-2009. a-e** spring (March-April-May: MAM). **f-j** summer (June-July-August: JJA). **k-o** autumn (September-October-November: SON). **p-t** winter (December-January-February: DJF). NTP, the northern Tibetan Plateau. This map was generated with The NCAR Command Language (Version 6.6.2) [Software]. (2019). Boulder, Colorado: UCAR/NCAR/CISL/TDD. http://dx.doi.org/10.5065/D6WD3XH5.

**Supplementary Table 1. Summary of sites where the IAE occurs.**

| Site name | Latitude (°) | Longitude (°) | Elevation (m, a.s.l) | Sampling type | Sampling season | Reference |
|---|---|---|---|---|---|---|
| Alps | 47.18 | 12.68 | 1400-3200 | Pack snow | — | [1] |
| Central Hindu Kush | 35.87 | 71.06 | 5000-5900 | Fresh snow | Summer | [2] |
| Nevado Illimani Sur | -16.65 | -67.78 | 5300-5700 | Fresh snow | Summer | [2] |
| Elbrus | 43.33 | 42.43 | 3000-4000 | Fresh snow | Winter, summer | [3] |
| Hulugou | 38.25 | 99.85 | 3400-4000 | Surface snow | Summer | [4] |
| Robertson Valley | 50.70 | -115.30 | 1980-2950 | Fresh snow | Spring, autumn, winter | [5] |
| Yulong | 27.17 | 100.25 | 3200-4000, 4550-4900 | Residual snow | Autumn | [6] |
| Ailao mountains | 23.08 | 102.75 | 1957-2024 | Meteoric water | Rainy season | [7] |
| Thailand | 13.04 | 101.50 | 0-600 | Meteoric water | Winter | [8] |
| Tianshan mountains | 43.50 | 87.63 | 2336-3805 | Meteoric water | Summer | [9] |
| Tianshan mountains | 43.28 | 87.18 | 918-3545 | Meteoric water | All seasons | [10] |
| Kayiertesi river | 47.58 | 89.75 | 1300-2900 | River water | Summer | [11] |
| Lake Ghirla | 45.92 | 8.82 | 442-1030 | River water | Spring | [12] |
| Satopanth Glacier | 31.25 | 79.37 | 4200-4600 | Channel water | Pre-monsoon | [13] |
| Wyoming | 41.34 | -106.31 | 2950-3250 | Stream water | — | [14] |
| Guliya | 35.28 | 81.48 | 6200-6700 | Ice core | — | [15] |
| Maxian | 35.80 | 104.00 | 2060-2800 | Leaf wax | Autumn | [16] |
| Xiying | 37.65 | 101.80 | 1750-2200 | Leaf wax | Autumn | [16] |
| Tianshan mountains | ~43 | ~87 | 3200-3800 | Leaf wax | Summer | [17] |

**Supplementary Table 2. Moisture contributions from different atmospheric levels over the moisture source regions to different levels over the target regions (the WUSA and the NTP) during the summer months.** The levels of the columns refer to the pressure levels of the TES $\delta D_v$ over the target regions, while the levels of the rows refer to the pressure levels of moisture contribution over the moisture source regions that follow the levels of the ERA reanalysis. The geographical distributions of trajectory starting positions of the WUSA and the NTP are shown in Supplementary Fig. 3. WUSA, western United States of America; NTP, the northern Tibetan Plateau.

| | | 1000 hPa | 900 hPa | 825 hPa | 750 hPa | 700 hPa | 600 hPa | 500 hPa | 400 hPa | Sum |
|---|---|---|---|---|---|---|---|---|---|---|
| WUSA | 825 hPa | 0.28 | 0.29 | 0.32 | 0.04 | 0.05 | 0.02 | 0.00 | 0.00 | 1 |
| | 750 hPa | 0.31 | 0.17 | 0.25 | 0.12 | 0.11 | 0.04 | 0.00 | 0.00 | 1 |
| | 681 hPa* | 0.23 | 0.11 | 0.15 | 0.14 | 0.27 | 0.08 | 0.02 | 0.00 | 1 |
| | 618 hPa* | 0.13 | 0.08 | 0.11 | 0.10 | 0.38 | 0.17 | 0.03 | 0.00 | 1 |
| | 510 hPa | 0.04 | 0.03 | 0.04 | 0.05 | 0.18 | 0.51 | 0.14 | 0.01 | 1 |
| NTP | 825 hPa | 0.23 | 0.28 | 0.23 | 0.13 | 0.10 | 0.03 | 0.00 | 0.00 | 1 |
| | 750 hPa* | 0.18 | 0.21 | 0.20 | 0.18 | 0.17 | 0.06 | 0.00 | 0.00 | 1 |
| | 681 hPa* | 0.14 | 0.18 | 0.16 | 0.13 | 0.27 | 0.10 | 0.02 | 0.00 | 1 |
| | 618 hPa* | 0.10 | 0.13 | 0.11 | 0.10 | 0.30 | 0.21 | 0.05 | 0.00 | 1 |
| | 510 hPa | 0.05 | 0.08 | 0.06 | 0.05 | 0.13 | 0.36 | 0.24 | 0.03 | 1 |

Note: * marks the levels where the IAE mainly occurs in summer over the WUSA and the NTP.

**Supplementary Table 3. Moisture contributions from different atmospheric levels over the moisture source regions to the target 618 hPa level over the WUSA and the NTP in different seasons.** MAM refers to March-April-May (spring season); JJA refers to June-July-August (summer season); SON refers to September-October-November (autumn season); DJF refers to December-January-February (winter season). WUSA, western United States of America; NTP, the northern Tibetan Plateau.

| | | 1000 hPa | 900 hPa | 825 hPa | 750 hPa | 700 hPa | 600 hPa | 500 hPa | 400 hPa | Sum |
|---|---|---|---|---|---|---|---|---|---|---|
| WUSA | MAM | 0.13 | 0.10 | 0.12 | 0.10 | 0.38 | 0.14 | 0.03 | 0.00 | 1 |
| | JJA* | 0.13 | 0.08 | 0.11 | 0.10 | 0.38 | 0.17 | 0.03 | 0.00 | 1 |
| | SON | 0.10 | 0.10 | 0.13 | 0.10 | 0.38 | 0.16 | 0.03 | 0.00 | 1 |
| | DJF | 0.14 | 0.14 | 0.16 | 0.11 | 0.31 | 0.12 | 0.02 | 0.00 | 1 |
| NTP | MAM* | 0.06 | 0.11 | 0.13 | 0.11 | 0.33 | 0.21 | 0.05 | 0.00 | 1 |
| | JJA* | 0.10 | 0.13 | 0.11 | 0.10 | 0.30 | 0.21 | 0.05 | 0.00 | 1 |
| | SON* | 0.05 | 0.08 | 0.09 | 0.10 | 0.35 | 0.26 | 0.07 | 0.00 | 1 |
| | DJF | 0.03 | 0.06 | 0.12 | 0.11 | 0.35 | 0.26 | 0.07 | 0.00 | 1 |

Note: * marks the seasons when the IAE mainly occurs at the 618 hPa level over the WUSA and the NTP.

## Supplementary references


1. Moser, H. & Stichler, W. Deuterium and oxygen-18 contents as an index of the properties of snow covers. Snow Mechanics Symposium, Proc. Grindelwald Symp. 114, pp. 122–135 (1974).

2. Niewodniczański, J., Grabczak, J., Barański, L. & Rzepka, J. The altitude effect on the isotopic composition of snow in high mountains. *J. Glaciol*. **27**, 99-111 (1981).

3. Vasil'chuk, Y. et al. Vasil'chuk, A variation of stable isotope composition of snow with altitude on the Elbrus mountain, Central Caucasus. *Geogr. Environ. Sustain*. **13**, 172-182 (2020).

4. Li, Z. et al. The stable isotope evolution in Shiyi glacier system during the ablation period in the north of Tibetan Plateau, China. *Quat. Int.* **380**, 262–271 (2015).

5. Moran, T. A., Marshall, S. J., Evans, E. C. & Sinclair, K. E. Altitudinal gradients of stable isotopes in lee-slope precipitation in the Canadian Rocky Mountains. *Arct. Antarct. Alp. Res.* **39**, 455–467 (2007).

6. He, Y. et al. Spatial and temporal variations of oxygen isotopes in snowpacks and glacial runoff in different types of glacial area in western China. *Ann. Glaciol*. **43**, 269-274 (2006).

7. Jiao, Y. et al. Impacts of moisture sources on the isotopic inverse altitude effect and amount of precipitation in the Hani Rice Terraces region of the Ailao Mountains. *Sci. Total Environ*. **687**, 470–478 (2019).

8. Laonamsai, J., Ichiyanagi, K., Kamdee, K., Putthividhya, A. & Tanoue, M. Spatial and temporal distributions of stable isotopes in precipitation over Thailand. *Hydrol. Processes* **35**, e13995 (2021).

9. Gu, W. & Longinelli, A. A case study on the hydrological significance of stable isotope data on alpine catchments with snow cover and glaciers, Xinjiang, China. *IAHS Publications-Publications of the Inter. Asso. Hydro. Sci.* **218**, 371-384 (1993).

10. Kong, Y. & Pang, Z. A positive altitude gradient of isotopes in the precipitation over the Tianshan Mountains: Effects of moisture recycling and sub-cloud evaporation. *J. Hydrol.* **542**, 222–230 (2016).

11. Zhang, W., Kang, S. C., Shen, Y. P., He, J. Q. & Chen, A. A. Response of snow hydrological processes to a changing climate during 1961 to 2016 in the headwater of Irtysh River Basin, Chinese Altai Mountains. *J. Mt. Sci.* **14**, 2295–2310 (2017).

12. Wirth, S. B. & Sessions, A. L. Plant-wax D/H ratios in the southern European Alps record multiple aspects of climate variability. *Quat. Sci. Rev.* **148**, 176-191 (2016).

13. Pant, N. et al. Tracing the isotopic signatures of cryospheric water and establishing the altitude effect in Central Himalayas: A tool for cryospheric water partitioning. *J. Hydrol.* **595**, 125983 (2021).

14. Miller, S. A., Mercer, J. J., Lyon, S. W., Williams, D. G. & Miller, S. N. Stable isotopes of water and specific conductance reveal complimentary information on streamflow generation in snowmelt-dominated, seasonally arid watersheds. *J. Hydrol.* **596**, 126075 (2021).

15. Thompson L. G. et al. Beaudon, K. Duan, M. R. Sierra-Hernandez, D. V. Kenny. Ice core records of climate variability on the Third Pole with emphasis on the Guliya ice cap, western Kunlun Mountains. *Quat. Sci. Rev.* **188**, 1-14 (2018).

16. Bai, Y., Tian, Q., Fang, X. & Wu, F. The “inverse altitude effect” of leaf wax-derived n-alkane δD on the northeastern Tibetan Plateau. *Org. Geochem.* **73**, 90–100 (2014).

17. Luo, P. et al. Empirical relationship between leaf wax n-alkane δD and altitude in the Wuyi, Shennongjia and Tianshan Mountains, China: Implications for paleoaltimetry. *Earth Planet. Sci. Lett.* **301**, 285–296 (2011).
